# UGCG influences glutamine metabolism of breast cancer cells

**DOI:** 10.1038/s41598-019-52169-7

**Published:** 2019-10-30

**Authors:** Nina Schömel, Sarah E. Hancock, Lisa Gruber, Ellen M. Olzomer, Frances L. Byrne, Divya Shah, Kyle L. Hoehn, Nigel Turner, Sabine Grösch, Gerd Geisslinger, Marthe-Susanna Wegner

**Affiliations:** 1 pharmazentrum frankfurt/ZAFES, Institute of Clinical Pharmacology, Johann Wolfgang Goethe University, Theodor Stern-Kai 7, 60590 Frankfurt am Main, Germany; 2 School of Medical Sciences, University of New South Wales, Sydney, New South Wales 2052 Australia; 3 School of Biotechnology and Biomolecular Sciences, University of New South Wales, Sydney, New South Wales 2052 Australia; 4 Fraunhofer Institute for Molecular Biology and Applied Ecology IME, Project Group Translational Medicine and Pharmacology (TMP), Theodor Stern-Kai 7, 60590 Frankfurt am Main, Germany

**Keywords:** Breast cancer, Cancer metabolism

## Abstract

*UDP-glucose ceramide glucosyltransferase* (UGCG) is the key enzyme in glycosphingolipid (GSL) metabolism by being the only enzyme that generates *glucosylceramide* (GlcCer) *de novo*. Increased UGCG synthesis is associated with pro-cancerous processes such as increased proliferation and multidrug resistance in several cancer types. We investigated the influence of UGCG overexpression on glutamine metabolism in breast cancer cells. We observed adapted glucose and glutamine uptake in a limited energy supply environment following UGCG overexpression. Glutamine is used for reinforced oxidative stress response shown by increased mRNA expression of glutamine metabolizing proteins such as *glutathione-disulfide reductase* (GSR) resulting in increased reduced glutathione (GSH) level. Augmented glutamine uptake is also used for fueling the *tricarboxylic acid* (TCA) cycle to maintain the proliferative advantage of UGCG overexpressing cells. Our data reveal a link between GSL and glutamine metabolism in breast cancer cells, which is to our knowledge a novel correlation in the field of sphingolipid research.

## Introduction

With about 2.1 million diagnosed cases in 2018, breast cancer is accountable for almost one in four cancer cases among women. Despite extensive research, breast cancer is the leading cause of cancer death in more than 100 countries and the most frequently diagnosed cancer^1^. Due to population aging, improved diagnostic methods and environmental changes, the incidence of breast cancer is predicted to increase^2^. Furthermore, *multidrug resistance* (MDR) development leads to cancer therapy failure. *UDP-glucose ceramide glucosyltransferase* (UGCG) is connected to MDR in several cancer types^3^. UGCG is the key enzyme in the *glycosphingolipid* (GSL) metabolism by transferring UDP-glucose to ceramide resulting in *glucosylceramide* (GlcCer) production. GlcCer is the precursor for all GSL and our previous studies showed that UGCG overexpression in MCF-7 cells leads to alterations in the composition of *glycosphingolipid-enriched microdomains* (GEMs) resulting in increased cell proliferation, although nutritional supply was restricted^4^. Rapid growth is one cancer cell hallmarks. Since enhanced proliferation entails increased demand for nutrients to serve as building blocks for macromolecules such as proteins, DNA, RNA and lipids, as well as the carbon source for metabolic energy generation, cancer cells have developed mechanisms to increase nutrient uptake. One major energy source for mammalian cells is glucose, which is amongst others an important substrate for protein and lipid synthesis (reviewed in^5^). Glucose uptake is accomplished by 14 different glucose transporters (GLUT1-14) (reviewed in^6^), whereas GLUT1 is considered as a major transporter of basal glucose uptake and is expressed ubiquitously in human tissue (reviewed in^5^). Tumor cells increase their glucose uptake and usage and shuttle glucose to alternative pathways as compared to normal cells (reviewed in^7^). The non-essential amino acid glutamine plays a special role in tumor cell metabolism. Although it can be generated endogenously from glucose-derived carbons and amino acid-derived ammonia, glutamine is known to be consumed by some cancer cells in an extensive amount. Glutamine fuels anaplerosis in the *tricarboxylic acid* (TCA) cycle and nucleotide and fatty acid biosynthesis (reviewed in^7^). The TCA cycle in the mitochondria is essential for cell energy metabolism, synthesis of macromolecules and sustaining redox balance (reviewed in^7^). Additionally, recent studies have shown that glutamine is also involved in lactate production, chromatin modification, facilitation of the transport of other amino acids and regulation of cell signaling (reviewed in^8^). However, Ta *et al*. showed that lactate is mainly produced from glucose carbons, whereas glutamine contributes only little to lactate production^9^. Glutamine is an important building block of glutathione (GSH) through transformation into glutamate. Glutamate can be further processed for synthesis of GSH, which is a primary cellular antioxidant tripeptide composed of glutamate, cysteine and glycine. Tumor cells persistently encounter high levels of *reactive oxygen species* (ROS) due to genetic, metabolic and microenvironment-related alterations. This is balanced for example by an increase in the antioxidant capacity of the cells. Following uptake glutamine can be used for glutamate production by *glutaminase* (GLS and GLS2) activity. Subsequently, the *glutamate-cysteine-ligase* (GCLC) produces ɣ-glutamylcysteine, which can be further metabolized by *glutathione synthase* (GSS) to GSH. The reduced GSH is the main thiol molecule in cells and can reduce protein disulfide bonds by serving as an electron donor^10^. The ratio of GSH to its oxidized form (GSSG) determines the redox state of the cell (reviewed in^11^). Activity of *glutathione reductase* (GSR) leads to production of reduced GSH derived from GSSG. Our previous results show an increased proliferation of UGCG overexpressing MCF-7 (MCF-7/UGCG OE) cells in an environment with reduced nutritional supply^4^. This was accompanied by doxorubicin resistance and induction of anti-apoptotic genes, which is presumably mediated by an altered composition of GEMs and AKT and ERK1/2 signaling pathway induction^4^. Knockdown of the UGCG or inhibiting the enzyme with DL-threo-1-phenyl-2-palmitoyl-amino-3-morpholino1-propanol (PPMP) abolished the effects^4^ and since these effects were prominent despite limited nutritional supply, we questioned whether or not the UGCG has an impact on the energy metabolism of breast cancer cells.

Here, we investigated the molecular mechanisms leading to the proliferation advantage in MCF-7/UGCG OE cells as compared to control cells. Our data show the strong effect of UGCG overexpression on glutamine uptake, which is augmented and used for a strongly increased glutamine oxidation and an increased oxidative stress response. The revealed cellular mechanisms give new insights into the role of the UGCG in cancer cell energy metabolism and may contribute to better understanding of cancer cell adaption to poor nutritional supply.

## Material and Methods

### Cell culture

The MCF-7 human breast adenocarcinoma cell line was purchased from the Health Protection Agency (European Collection of Cell Cultures EACC, Salisbury, UK). Cells were cultured at 37 °C in an atmosphere containing 5% CO_2_ in Dulbecco’s Modified Eagle Medium (DMEM), which contains a high glucose level (4500 mg/L-glucose), no HEPES, no phenol-red, 5% charcoaled *fetal bovine serum* (FBS), 1% GlutaMAX and 1% sodium pyruvate (Sigma-Aldrich, St. Louis, Missouri, USA). Stably transfected cells were selected by the supplementation of 200 µg/ml G418 (Thermo Fisher Scientific, Waltham, USA).

### Generation of stable UGCG expressing cells

Cells were stably transfected with the UGCG expression plasmid (pCMV6-ENTRY vector, OriGene Technologies Inc., Rockville, USA) (=MCF-7/UGCG OE) using Lipofectamine 2000 (Invitrogen, Carlsbad, USA) and selected with G418 as previously described^4^. As a control, MCF-7 cells expressing the pCMV-HA-tag vector (=MCF-7/empty) (generous gift from Dr. Manuel Kaulich, Institute of Biochemistry II, Johann Wolfgang Goethe University, Frankfurt am Main, Germany) were established and selected with G418.

### Determination of the intracellular glutamine concentration

The glutamine concentration in MCF-7 cells was determined using the EnzyChrom Glutamine Assay Kit (EGLN-100, BioAssay Systems, Hayward, USA). All steps were performed according to the manufacturer’s protocol.

### Analysis of intracellular glucose concentration

To determine intracellular glucose level, the Picoprobe Glucose Assay Kit (ab169559, Abcam, Cambridge, UK) was used according to the manufacturer’s protocol. Briefly, 1 × 10^6^ cells were lysed, a reaction mix was added, and the fluorescence was measured at Ex/Em 535/587 nm after 30 min of incubation. Prior to the assay, the samples were deproteinized using 1 M perchloric acid and neutralized by adding potassium hydroxide. The guidance of the measurement by Tanja Wotapek (research group Dr. Thomas Oellerich, Department of Medicine, Hematology/Oncology, Goethe University, Frankfurt, Germany, Director: Prof. Dr. med. Hubert Serve) is gratefully acknowledged.

### Proliferation assay

5 × 10^4^ cells per 6-well were seeded in control medium (see section *Cell Culture*) or without glutamine supplementation (GlutMAX) or in DMEM with low glucose (1000 mg/L-glucose) (day 0). Cells were harvested on day 1, 2, 3 and 4 and the living cell number determined by using trypan blue staining and the Neubauer counting chamber.

### Quantitative real-time PCR (qRT-PCR)

Total RNA was isolated using the RNeasy Mini Kit (Quiagen, Hilden, Germany) according to the manufacturer’s protocol. To determine RNA concentration, the absorption at 260 nm was measured using the Infinite 200 NanoQuant (Tecan Group, Maennedorf, Switzerland). 300 ng RNA were used to perform cDNA synthesis with the Verso cDNA Synthesis Kit (Thermo Fisher Scientific, Waltham, USA). Gene-specific PCR products were detected using 5X QPCR Mix EvaGreen (ROX) (Bio&Sell, Feucht, Germany) on a QuantStudio 5 Real-time PCR System (Thermo Fisher Scientific, Waltham, USA). Relative mRNA expression was calculated according to the Δct method and normalized to the expression of the housekeeping gene *60 S ribosomal protein L*3*7a* (RPL37A). Primer, as listed in Table 1, for GluR1, GLUD, xCt, Lat1, Aralar, GLUT1, 8, 10, 11, 12, and 13 were purchased from Eurofins (Luxembourg, Luxembourg) and the primer for RPL37A were synthesized by BioSpring (Frankfurt am Main, Germany). The primer sets for GCLC, GLS, GLS2, GSR, GSS, NFE2L2 and were purchased from RealTime Primers (Elkins Park, USA). The GLUT6 primer was purchased from Sigma-Aldrich (St. Louis, Missouri, USA) and ACC1 and ACC2 primer were purchased from Integrated DNA Technologies (Coralville, Iowa, USA).

**Table 1:** qRT-PCR primer sequences.

Gene	Forward primer 5′ → 3′	Reverse primer 5′ → 3′	Amplicon (bp)
RPL37A	attgaaatcagccagcacgc	aggaaccacagtgccagatcc	94
GLS	ctacaggattgcgaacgtct	acctttcctccagactgctt	244
GLS2	aggcattccgaaagaagttt	gtcagtgcctagggtgctta	272
GSR	cccacaatagaggtcagtgg	caatgtaacctgcaccaaca	184
GSS	acagctgaaggacagtgagg	gagtgtcttttcctgcctga	166
GCLC	agttgaggccaacatgcgaa	catctccaccaacacagaca	182
NFE2L2	caactccaaaaggagcaaga	aaacgtagccgaagaaacct	240
ASCT2	gtgttcattgcacagctcag	acattgaggacggtacagga	221
xCT	ggtccattaccagcttttgtacg	aatgtagcgtccaaatgccag	96
Lat1	gtggacttcgggaactatcacc	gaacagggacccattgacgg	96
Aralar	tcaaggtgcagacaactaagc	ggggtcatataacgctctcca	100
GLUD	gaagctgcggcttaaaaggg	tagcggtacatggccacaag	125
GLUT 1	attggctccggtatcgtcaac	gctcagataggacatccagggta	174
GLUT 6	gcccggactacgacacct	agctgaaattgccgagcac	126
GLUT 8	ccggcatctacaagcccttc	atagaacatgacggcgttgac	158
GLUT 10	cttgctgtatctacgtgtcagag	ccagccagtgcatagttgagg	124
GLUT 11	ggagtcaatgcaggtgtgag	ccagagccgtaaagatggctg	162
GLUT 12	aacatgcggacccgaataatg	aatgaccttgacgactccaac	168
GLUT 13	acattgcggaggtctcacc	aggctccatcaacaacacttg	105
ACC1	atcccgtaccttcttctactg	cccaaacataagccttcactg	164
ACC2	cggatgcgtaacttcgatctg	ctatggtccgtcacttccacac	107

### Isolation and identification of glycosphingolipid-enriched microdomains (GEMs)

GEMs of the cell membranes of MCF-7 cells were isolated and identified by determination of the cholesterol level, GSL level and raft marker caveolin-1 detection as previously described in^4^. Briefly, cells were harvested, processed and sonified in MES buffer and ultra-centrifuged in a saccharose density gradient. Ten fractions were isolated and purified from saccharose and the GEM containing fractions were identified. To determine the level of the glutamine transporter ASCT2 in the fractions, the purified fractions were analyzed as described in the section *Determination of protein concentration by Western blot analysis*.

### Determination of protein concentration by western blot analysis

For ASCT2 protein concentration analysis, fractions were generated by saccharose density gradient centrifugation as described in^4^. Protein concentration was determined via the Bicinchoninic acid method. 60 µg total protein extract were separated by 10% SDS-PAGE and electro-blotted onto a nitrocellulose membrane (Amersham Protran, GE Healthcare Life Sciences, Freiburg, Germany). Protein transfer was verified by Ponceau S staining (0.5% in 1% acetic acid). After 90 minutes incubation in Odyssey Blocking Buffer (LI-COR Biosciences, Bad Homburg, Germany) 1:1 diluted in PBS with 0.1% Tween 20 (PBST), the membrane was incubated with the primary antibody anti-ASCT2 (Abcam, Cambridge, UK). The membrane was washed twice with PBST and incubated with an IRDye 680 conjugated secondary antibody (LI-COR Biosciences, Bad Homburg, Germany). The Odyssey Infrared Scanner (LI-COR Biosciences, Bad Homburg, Germany) was used for fluorescence emission analysis and densitometric analysis was performed with the Image Studio Lite software (LI-COR Biosciences, Bad Homburg, Germany). For ACC protein concentration determination 1 × 10^6^ cells were lysed in 250 μl RIPA buffer (150 mM NaCl, 1% NP-40, 0.5% Na-dCla, 0.1% SDS, 50 mM Tris, distilled water, protease inhibitor) on ice for 10 mins. Subsequently, samples were centrifuged for 30 min at 16,000 g and 4 °C. The protein containing supernatant was transferred into a fresh Eppendorf tube. Following protein separation by 7.5% SDS-PAGE and electro-blotting, membranes were incubated with an anti-ACC total antibody (Cell Signaling, Danvers, Massachusetts, USA), an anti-phospho ACC (Ser79) antibody and Hsp90 antibody (BD Biosciences, Franklin Lakes, New Jersey, USA). The membrane was washed twice with PBST and incubated with an IRDye 680 and IRDye 800 conjugated secondary antibody (LI-COR Biosciences, Bad Homburg, Germany). The Odyssey Infrared Scanner (LI-COR Biosciences, Bad Homburg, Germany) was used for fluorescence emission analysis and densitometric analysis was performed with the Image Studio Lite software (LI-COR Biosciences, Bad Homburg, Germany).

### Determination of total ROS levels

For intracellular total ROS level determination, a ROS assay using *5-(and-6)-chloromethyl-2*′,*7*′*-dichlorodihydrofluorescein diacetate, acetyl ester* (CM-H_2_DCFDA) (Invitrogen, Carlsbad, USA) was performed according to manufacturer’s protocol. Following passive diffusion into cells the acetate groups of CM-H_2_DCFDA are cleaved by esterases and the thiol-reactive chloromethyl group reacts with GSH and other thiols. Subsequent oxidation yields a fluorescent adduct that is trapped inside the cell and is measured at Ex/Em ~492–495/517–527 nm.

### Determination of GSH and GSSG levels and GSH/GSSG ratio calculation

For intracellular glutathione (reduced (GSH) and oxidized (GSSG)) concentration the GSH Fluorometric Assay Kit (BioVision, Zurich, Switzerland) was used. Briefly, 1 × 10^6^ cells were homogenized and the assay performed according to manufacturer’s protocol.

### Determination of the total antioxidant capacity

The colorimetric Total Antioxidant Capacity Assay Kit (ab65329, Abcam, Cambridge, UK) was used to quantify the antioxidant capacity of MCF-7 cells. The assay was conducted according to the manufacturer’s protocol. In brief, cells of one 80% confluent T75 flask were harvested and lysed using ddH_2_O. A Cu^2+^ solution was added and Cu^2+^ ions were converted to Cu^+^ by both small molecule and protein antioxidants. The reduced Cu^+^ ions were chelated with a colorimetric probe giving a broad absorbance peak around OD 570 nm, which is proportional to the total antioxidant capacity. The absorbance at 562 nm was measured using the Infinite 200 NanoQuant (Tecan Group, Maennedorf, Switzerland) and samples were related to a trolox standard curve. The fold increase of MCF-7/UGCG OE compared to MCF-7/empty values was indicated.

### Determination of NADPH and NADP^+^ levels and NADPH/NADP^+^ ratio calculation

For intracellular NADPH and NADP^+^ concentration determination the NADP/NADPH-Glo Assay (Promega, Madison, USA) was used. Briefly, 20,000 cells were seeded in a clear 96-well plate. The assay was performed according to manufacturer’s protocol.

### Metabolomics by LC-MS

Untargeted metabolomics analysis was performed by using the method described by Mackay *et al*.^12^. For steady-state labelling experiments, cells were plated in triplicate from three different passages in 6-well plates and allowed to adhere overnight. Cells were then switched to media containing 2 mM uniformly-labelled 13C5 L-glutamine, and metabolites were extracted after 16 hours. After removal of the media cells were washed twice with 500 μl of ice-cold PBS to quench their metabolism and ice-cold extraction solvent Methanol:Acetonitrile:MilliQ (50:30:20 v/v) was added at a rate of 1 ml per 2 × 10^6^ cells. Cells were scraped and transferred into an Eppendorf tube. Blank wells (i.e. no cells) for background subtraction were processed in the same fashion. Samples were incubated on a rotator at 4 °C for 10 minutes. Subsequently, samples were centrifuged at 16,100 x g for 10 minutes at 4 °C and the same amount of supernatant in a glass vial transferred to a new vial. The pellet was used for protein concentration determination and supernatant was stored at −80 °C prior to analysis. For the LC-MS method a 150 mm × 2.1 mm, 5 μm ZIC pHILIC column was used (Merck, VIC, Australia). The aqueous mobile phase solvent was 20 mM ammonium carbonate, adjusted to pH 9.4 with 0.1% ammonium hydroxide solution (25%) and the organic mobile phase was 100% acetonitrile. A linear gradient from 80% organic to 80% aqueous was run over a 17 minute period, followed by an equilibration back to the starting condition. The flow rate used was 200 μl/min and the column temperature was 45 °C. Samples were maintained at 4 °C in the autosampler, prior to injection (4 µl). Metabolites were detected by high resolution mass analyzer (Q Exactive Plus, Thermo Fisher Scientific, VIC, Australia) using settings described previously^12^. Metabolite identification was confirmed by comparison with standards. Isotope labelling patterns of metabolites were analyzed by Xcalibur (v3.2, Thermo Fisher Scientific, VIC, Australia). Mass isotopomer correction was performed to correct for naturally occurring carbon isotopes.

### Immunohistochemistry of FFPE breast cancer tissue

Formalin-fixed paraffin-embedded (FFPE) human breast carcinoma and non-tumor controls were obtained from the Senckenberg Institute for Pathology of the Johann Wolfgang Goethe University (Frankfurt am Main, Germany). Slides were baked for one hour at 56 °C and rehydrated according to Table 2.

**Table 2:** Rehydration protocol for FFPE breast carcinoma tissue.

Substance	Incubation
xylene	15 min
xylene	10 min
96% ethanol	5 min
80% ethanol	5 min
70% ethanol	5 min
ddH_2_O	1 min (floating water)
ddH_2_O	1 min

After an incubation of 10 minutes in 10 mM citrate buffer (pH 6), slides were heated in the buffer for 3 × 5 minutes in the microwave at 800 watts. When cooled down, slides were blocked with 5% goat serum and 1% bovine serum albumin (BSA) in PBS. The primary anti-ASCT2 antibody (Abcam, Cambridge, UK) was incubated 1:200 in PBS containing 2% goat serum and 1% BSA over night at 4 °C. The secondary antibody anti-rabbit IgG (whole molecule), F(ab′)2 fragment–Cy3 antibody produced in sheep (Sigma-Aldrich, St. Louis, Missouri, USA) (dilution: 1:800 in PBS containing 2% goat serum and 1% BSA) was incubated for 60 minutes. Subsequently, DNA was stained using 4′,6-diamidino-2-phenylindole (DAPI) and the tissue was examined with an Axio Observer.Z1 microscope (Carl Zeiss AG; Oberkochen, Germany).

### Statistical analysis

Statistical analysis was performed with GraphPad Prism 7 software. Data are presented as mean ± standard error of the mean (SEM). Significant differences in means between two groups were assessed by unpaired *t* test with or without Welch’s correction as indicated in the figure description.

### Ethical approval

This article does not contain any studies with human participants or animals performed by any of the authors.

## Results

### UGCG overexpression leads to glutamine dependency

**Figure 1:**
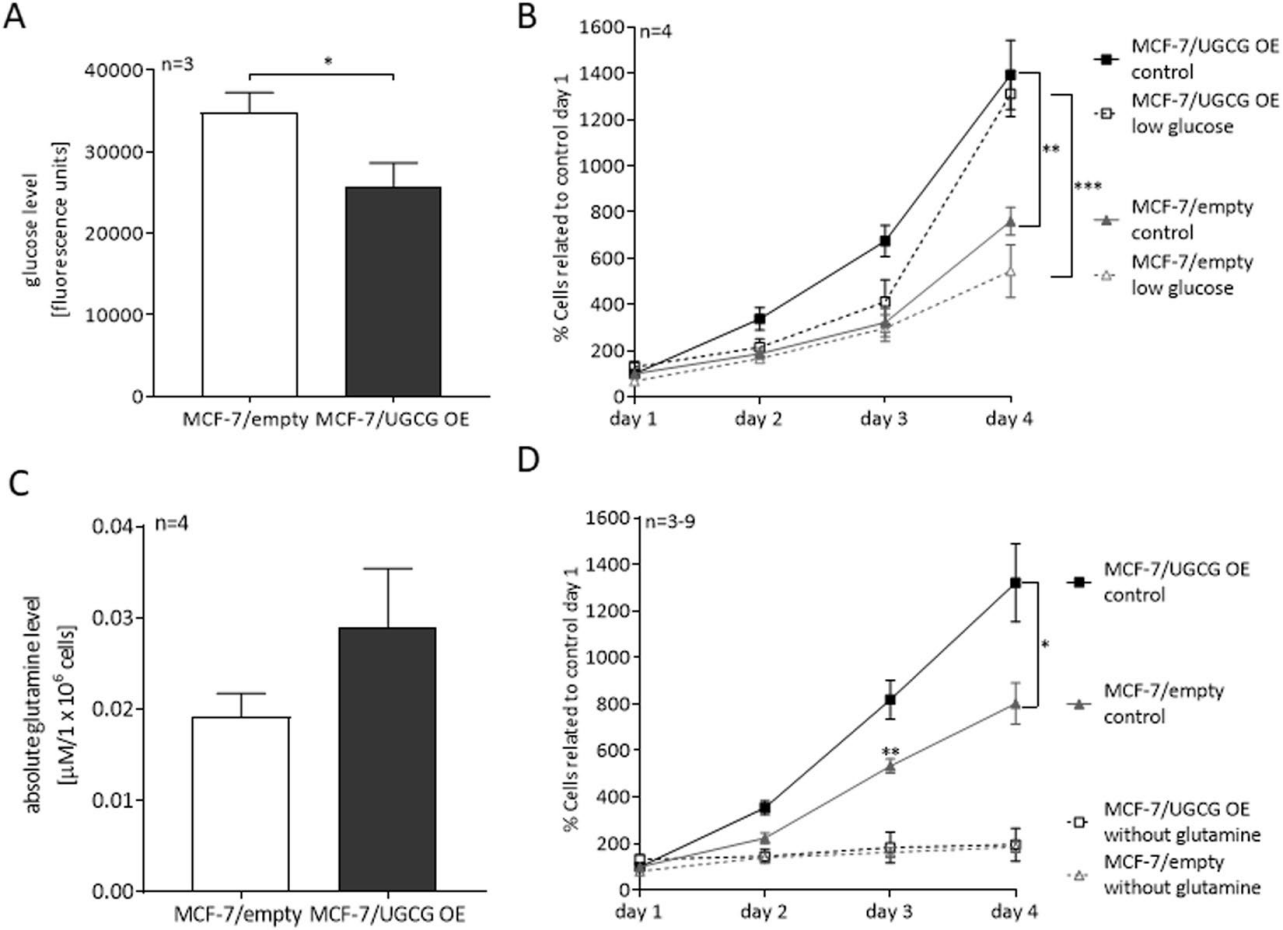
Analysis of intracellular glucose and glutamine levels and proliferation in glucose or glutamine deprived environment of MCF-7 cells. (**A**) Intracellular glucose level determined by a fluorescence based assay. Data are presented as a mean of *n* = 3 ± SEM. Unpaired *t* test with Welch’s correction. (**B**) Cell proliferation analysis under control (4500 mg/l glucose) and low glucose (1000 mg/l glucose) concentration. Data are presented as a mean of *n* = 4 ± SEM. Unpaired *t* test with Welch’s correction. (**C**) Absolute glutamine level quantified by a colorimetric assay. Data are presented as a mean of *n* = 4 ± SEM. (**D**) Cell proliferation under control glutamine supplementation (1% GlutaMax) and without glutamine supplementation. Data are presented as a mean of *n* = 3–9 ± SEM. Unpaired *t* test with Welch’s correction. *p ≤ *0*.*05*, **p ≤ *0*.*01*, ***p ≤ *0*.*001*.

Since glucose is an important energy source for cells and cancer cells are known for upregulating their glucose uptake, we measured the intracellular glucose concentration of MCF-7/UGCG OE and control cells. Surprisingly, MCF-7/UGCG OE cells exhibit a decreased intracellular glucose concentration as compared to control cells (Fig. 1A). To evaluate the direct influence of glucose availability in the environment on the advantage of MCF-7/UGCG OE cell proliferation, we performed proliferation assays under control and reduced glucose concentrations. MCF-7/UGCG OE cells exhibit an increased proliferation as compared to control cells (Fig. 1B). In addition, the proliferative advantage of MCF-7/UGCG OE cells is sustained in low-glucose medium indicating that the increased proliferation is not limited by decreased glucose supply (Fig. 1B). Because glutamine is a versatile nutrient, we also determined its intracellular concentration in MCF-7/UGCG OE and control cells. No statistically significant difference of intracellular glutamine levels between MCF-7/UGCG OE as compared to control cells could been shown (Fig. 1C). The proliferation assay results show that glutamine is essential for the proliferation advantage of MCF-7/UGCG OE cells (Fig. 1D) indicating a glutamine dependency of breast cancer cells following UGCG overexpression.

### UGCG overexpression influences mRNA expression of glucose transporters, glutamine metabolizing enzymes and in oxidative stress response involved genes

**Figure 2:**
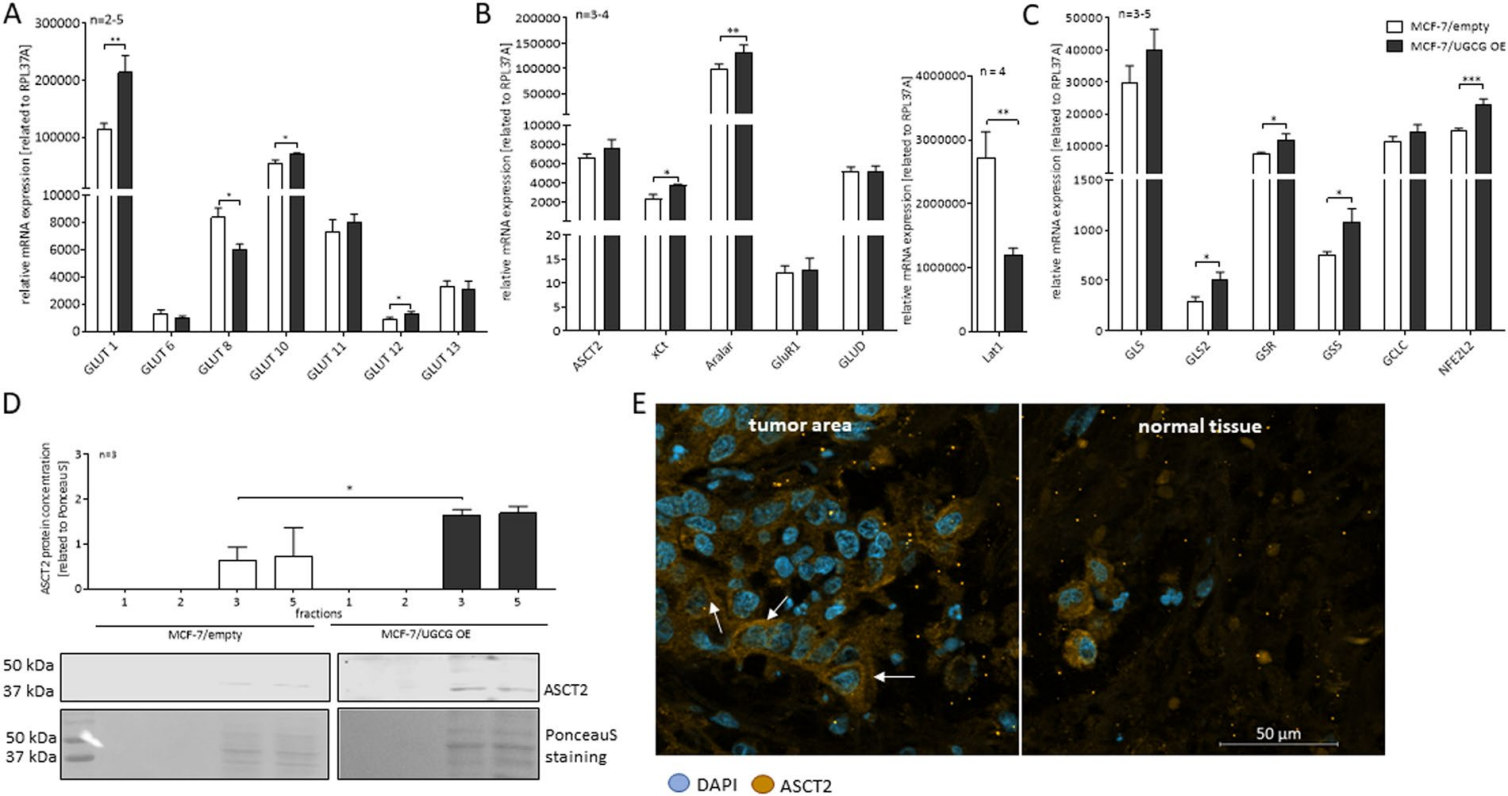
Analysis of mRNA expression of glucose transporters and glutamine metabolizing enzymes by qRT-PCR and ASCT2 translocation in MCF-7 cells. (**A**) mRNA expression analysis of *glucose transporters* (GLUT) 1, 6, 8, 10, 11, 12 and 13 related to the housekeeping gene RPL37A. Data are presented as a mean of *n* = 2–5 ± SEM. Unpaired *t* test with Welch’s correction. (**B**) mRNA expression analysis of the transporters *alanine-serine-cysteine transporter 2* (ASCT2), *glutamate/cystine antiporter* (xCT), *mitochondrial aspartate-glutamate-carrier* (Aralar), *glutamate carrier* (GluR1), *glutamate dehydrogenase* (GLUD) and *L-type amino acid transporter 1* (Lat1) related to the housekeeping gene RPL37A. Data are presented as a mean of *n* = 3–4 ± SEM. Unpaired *t* test with Welch’s correction. (**C**) mRNA expression analysis of the GSH synthesis key enzymes *glutaminase* (GLS) and GLS2, *glutathione reductase* (GSR), *glutathione synthase* (GSS), *glutamate-cysteine-ligase* (GCLC) and the transcription factor *Nuclear factor (erythroid-derived 2)-like 2* (NFE2L2) related to the housekeeping gene RPL37A. Data are presented as a mean of *n* = 3–5 ± SEM. Unpaired *t* test with Welch’s correction. (**D**) *Glycosphingolipid-enriched microdomains* (GEMs) were isolated by sucrose density gradient centrifugation and GEM containing fractions 2 and 3 were identified by cholesterol and caveolin-1 content (data not shown,^4^). The ASCT2 protein concentration of the fractions was determined by Western blot analysis and related to Ponceau S staining. One representative blot of three is displayed. Data are presented as a mean of *n* = 3 ± SEM. Unpaired *t* test with Welch’s correction. (**E**) Immunohistochemistry of formaldehyde-fixed paraffin-embedded (FFPE) human breast cancer tissue surrounded by non-cancerous tissue. Sections were incubated with an anti-ASCT2 antibody and 4′,6-diamidino-2-phenylindole (DAPI) was used to stain DNA. Images were recorded by Axio ObserverZ.1 microscope (Carl Zeiss AG, Oberkochen, Germany). *p ≤ *0*.*05*, **p ≤ *0*.*01*, ***p ≤ *0*.*001*.

Since MCF-7/UGCG OE cells were able to sustain increased proliferation following glucose deprivation, we analyzed the mRNA expression of the most abundant glucose transporters in MCF-7 cells. MCF-7/UGCG OE cells exhibit a significantly increased mRNA expression of GLUT1, GLUT10 and GLUT12, decreased mRNA expression of GLUT8, whereas mRNA expression of GLUT6, GLUT11 and GLUT13 was unaltered (Fig. 2A). Additionally, we analyzed the mRNA expression of several glutamine or glutamate transporters, because glutamine promotes cancer cell proliferation. mRNA expression of the *cystine glutamate antiporter* (xCT) was significantly increased following UGCG overexpression (Fig. 2B). The transporter xCT executes the exchange of intracellular glutamate with extracellular cysteine, which is an essential component in GSH synthesis. mRNA expression of the *L-type amino acid transporter 1* (Lat1), which transports essential amino acids such as leucine in exchange with glutamine into the cell, is significantly downregulated in MCF-7/UGCG cells as compared to control cells (Fig. 2B). mRNA expression of the most important glutamine transporter *alanine-serine-cysteine transporter 2* (ASCT2) was not altered. Interestingly, the *mitochondrial glutamate carrier* GluR1 was unchanged, but mRNA expression of the mitochondrial *aspartate glutamate carrier* Aralar was upregulated (Fig. 2B) suggesting a more important role in the context of UGCG overexpression for this transporter as compared to GluR1. Expression of the mitochondrial *glutamate dehydrogenase* (GLUD), which deaminates and oxidizes glutamate to *α-ketoglutarate* (α-KG), was unchanged following UGCG overexpression (Fig. 2B). Since glutamine seems to be important for MCF-7/UGCG OE cells, we analyzed mRNA expression of glutamine metabolizing and in oxidative stress response involved genes. The enzymes that convert glutamine to glutamate, are *glutaminase* (GLS) and its isoform *glutaminase 2* (GLS2). GLS, which is expressed to a much greater extent than GLS2, is increased, but not statistically significant in MCF-7/UGCG OE cells, whereas GLS2 mRNA expression is significantly increased (Fig. 2C). Following the pathways of glutamate in the cell, we further analyzed the expression of key enzymes of GSH synthesis. Strikingly, mRNA expression of *glutamate-cysteine-ligase* (GCLC), *glutathione synthase* (GSS) and *glutathione reductase* (GSR) are increased in MCF-7/UGCG OE cells as compared to control cells (Fig. 2C). mRNA expression of the transcription factor *nuclear factor (erythroid-derived 2)-like 2* (NFE2L2), which regulates GCLC, GSS and GSR gene transcription, was also increased following UGCG overexpression (Fig. 2C). The data indicate that UGCG overexpression leads to a more efficient uptake of glucose and glutamine and that glutamine is further metabolized.

### UGCG overexpression leads to ASCT2 translocation to GEMs in the plasma membrane

ASCT2 is the most important glutamine carrier for epithelial cells. Our previous studies revealed an altered GEM composition in MCF-7/UGCG OE cells as compared to control cells^4^. Since ASCT2 mRNA expression in MCF-7/UGCG OE cells as compared to control cells is unchanged, we analyzed the ASCT2 protein concentration in GEMs by Western blot analysis. The GEM containing fractions were determined by cholesterol level and caveolin-1 protein concentration as shown in our previous data^4^. A significantly increased amount of ASCT2 protein in the GEMs containing fraction 3 was detected in MCF-7/UGCG OE cells as compared to control cells (Fig. 2D and Supplemental Data 1) indicating that UGCG overexpression leads to translocation of ASCT2 into GEMs. We could also show this by breast cancer tissue staining, which shows that in tumorous tissue areas ASCT2 is mainly located in the plasma membrane (Fig. 2E).

### Increased oxidative stress response following UGCG overexpression

**Figure 3:**
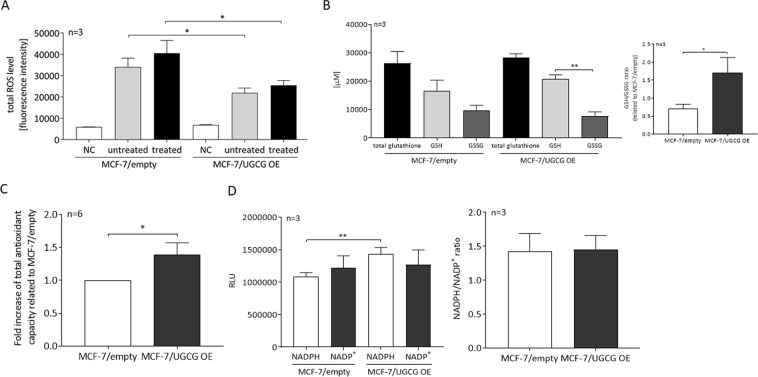
Analysis of the redox state of MCF-7 cells. (**A**) The total level of *reactive oxygen species* (ROS) was analyzed by a fluorescence-based assay. NC = negative control (no CM-H_2_DCFDA), untreated = 0 μM (*tert-butyl hydroperoxide* = TBHP), treated = 50 μM TBHP. TBHP induces oxidative stress via several mechanisms. Data are presented as mean of *n* = 3 ± SEM. Unpaired *t* test with Welch’s correction. (**B**) Reduced glutathione (GSH) and oxidized glutathione disulfide (GSSG) levels were measured using a glutathione fluorometric assay kit. The ratio of GSH/GSSG was calculated. Data are presented as a mean of *n* = 3 ± SEM. (**C**) The antioxidant capacity was quantified using a colorimetric assay. Trolox was used to standardize antioxidants. Data are presented as a mean of *n* = 6 ± SEM. Unpaired *t* test. (**D**) Determination of reduced (NADPH) and oxidized (NADP^+^) form of Nicotinamide adenine dinucleotide phosphate by a luminescence-based assay. Data are presented as a mean of *n* = 3 ± SEM. Unpaired *t* test with Welch’s correction. RLU = relative luminescent units. *p ≤ *0*.*05*, **p ≤ *0*.*01*.

**Figure 4:**
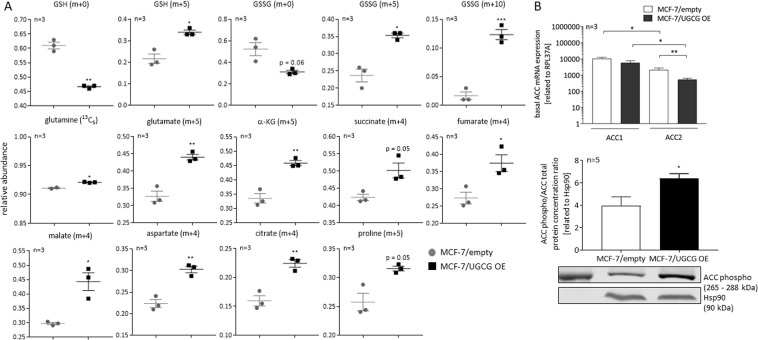
Analysis of metabolic flux of glutamine by ^13^C_5_-L-glutamine supplementation and subsequent LC-MS analysis. (**A**) Relative abundance of GSH, GSSG and TCA cycle metabolites was determined by using LC-MS analysis with ZIC-pHILIC chromatography. Data are presented as a mean of *n* = 3 ± SEM. Unpaired *t* test. (**B**) mRNA expression and protein concentration analysis of the *acetyl-CoA-carboxylase* (ACC). Basal ACC1 and ACC2 mRNA expression is determined by qRT-PCR and is related to the housekeeping gene RPL37A. Data are presented as a mean of *n* = *3* ± SEM. Unpaired *t* test with Welch’s correction. The ACC phospho/ACC total ratio protein concentration was determined by Western blot analysis using an anti-ACC total and anti-phospho-ACC antibody and is related to the housekeeper protein Hsp90. A representative blot is displayed. Data are presented as mean of *n* = 5 ± SEM. *p ≤ *0*.*05*, **p ≤ *0*.01,***p ≤ 0.001.

Since mRNA expression of genes involved in the oxidative stress response is increased, we determined the total ROS level. MCF-7/UGCG OE cells exhibit a significantly reduced total ROS level as compared to control cells (Fig. 3A). Even following treatment with *tert-butyl hydroperoxide* (TBHP) (5, 30 and 60 minutes), which induces oxidative stress by lipid peroxidation of membrane phospholipids and GSH depletion, MCF-7/UGCG OE cells exhibit a lower total ROS level as compared to control cells (Fig. 3A). Because MCF-7/UGCG OE cells seem to exhibit an increased capacity for ROS defense, we determined total glutathione, reduced (GSH) and oxidized glutathione (GSSG) levels. UGCG overexpression leads to a significant difference between GSH and GSSG levels, which is not present in control cells (Fig. 3B). Respectively, UGCG overexpression leads to an increased GSH/GSSG ratio (Fig. 3B), which indicates an increased potential to capture ROS of MCF-7/UGCG OE cells as compared to control cells and could be the reason for the reduced effectiveness of TBHP in MCF-7/UGCG OE cells. The data are in line with increased mRNA expression of the enzyme GSR, which reduces GSSG to GSH. To investigate the flux of glutamine to GSH and GSSG, we performed a ^13^C_5_-glutamine tracing assay. Unlabelled (m + 0) GSH is significantly decreased, whereas fully labelled GSH (m + 5) is increased in MCF-7/UGCG OE cells as compared to control cells (Fig. 4A). Unlabelled GSSG is decreased by tendency and half-labelled (m + 5) and fully labelled (m + 10) GSSG is significantly increased following UGCG overexpression (Fig. 4A). The data indicate a flux of glutamine to GSH and GSSG production, which is also indicated by upregulated mRNA expression of glutamine metabolizing enzymes. To confirm the data of a putative increased antioxidant potential following UGCG overexpression, the total antioxidant capacity was determined. The assay allows measuring the concentration of small molecules and protein antioxidants by conversion of Cu^2+^ ions to Cu^+^. MCF-7/UGCG OE cells exhibit an elevated antioxidant capacity as compared to control cells (Fig. 3C), which verifies the increased mRNA expression of GSH synthesizing enzymes. Furthermore, the intracellular concentration of NADPH (reduced) is increased following UGCG overexpression, whereas NADP^+^ (total oxidized) levels are unchanged (Fig. 3D). The NADPH/NADP^+^ ratio is not significantly altered (Fig. 3D). Our results show that UGCG overexpression leads to increased ROS defense mechanisms in breast cancer cells.

### Glutamine oxidation is increased following UGCG overexpression

Since glutamine synthesizing enzymes are increased in MCF-7/UGCG OE cells, which results in increased cell proliferation, we analyzed steady-state labelling of TCA cycle metabolites by ^13^C_5_-glutamine tracing assay. Our data reveal increased glutaminolysis by a significant increase in the amount of uniformly ^13^C-labelled glutamate (m + 5) detected in MCF-7 cells following UGCG overexpression (Fig. 4A and Supplemental Data 2). Furthermore, uniformly-labelled α-KG (m + 5), succinate (m + 4) (p = 0.05), fumarate (m + 4), malate (m + 4) and citrate (m + 4) level are increased (Fig. 4A), which indicates increased oxidation of glutamate and subsequent TCA cycle intermediates in MCF-7/UGCG OE cells. Since the citrate (m + 5) and acetyl-CoA (m + 2) level are unaltered (data not shown) as well as the aspartate (m + 3), fumarate (m + 3) and malate (m + 3), UGCG overexpression does not have an impact on the reductive metabolism. Furthermore, malate is not shuttled into the pyruvate cycle indicated by unaltered pyruvate (m + 3), acetyl-CoA (m + 2) and citrate (m + 6) level (data not shown). Accordingly, no evidence for changed malic enzyme activity is shown following UGCG overexpression. To investigate the contribution of oxidized glutamine to fatty acid synthesis we determined ACC1 and 2 mRNA and total protein expression. Interestingly, ACC1 mRNA expression is unchanged and ACC2 mRNA expression significantly decreased (Fig. 4B). ACC phospho/ACC total ratio protein concentration is increased in MCF-7/UGCG OE cells (Fig. 4B and Supplemental Data 3) indicating decreased ACC enzyme activity. Our data indicate that glutamine is extensively oxidized in UGCG overexpressing cells and therefore, contributes to the coverage of the high energy demand of MCF-7/UGCG OE cells.

### Glutamine may serve as a precursor for amino acid synthesis

The ^13^C_5_-glutamine tracing assay data show increased level of aspartate (m + 4) following UGCG overexpression (Fig. 4A). Aspartate may function as a precursor for asparagine, whereas no difference in asparagine (m + 4) level between MCF-7/UGCG OE and control cells could be detected (data not shown). In addition, the proline (m + 5) level is increased (p = 0.05) as well in MCF-7/UGCG OE as compared to control cells, indicating increased generation of proline from glutamine via glutamate in MCF-7/UGCG OE cells (Fig. 4A).

## Discussion

**Figure 5:**
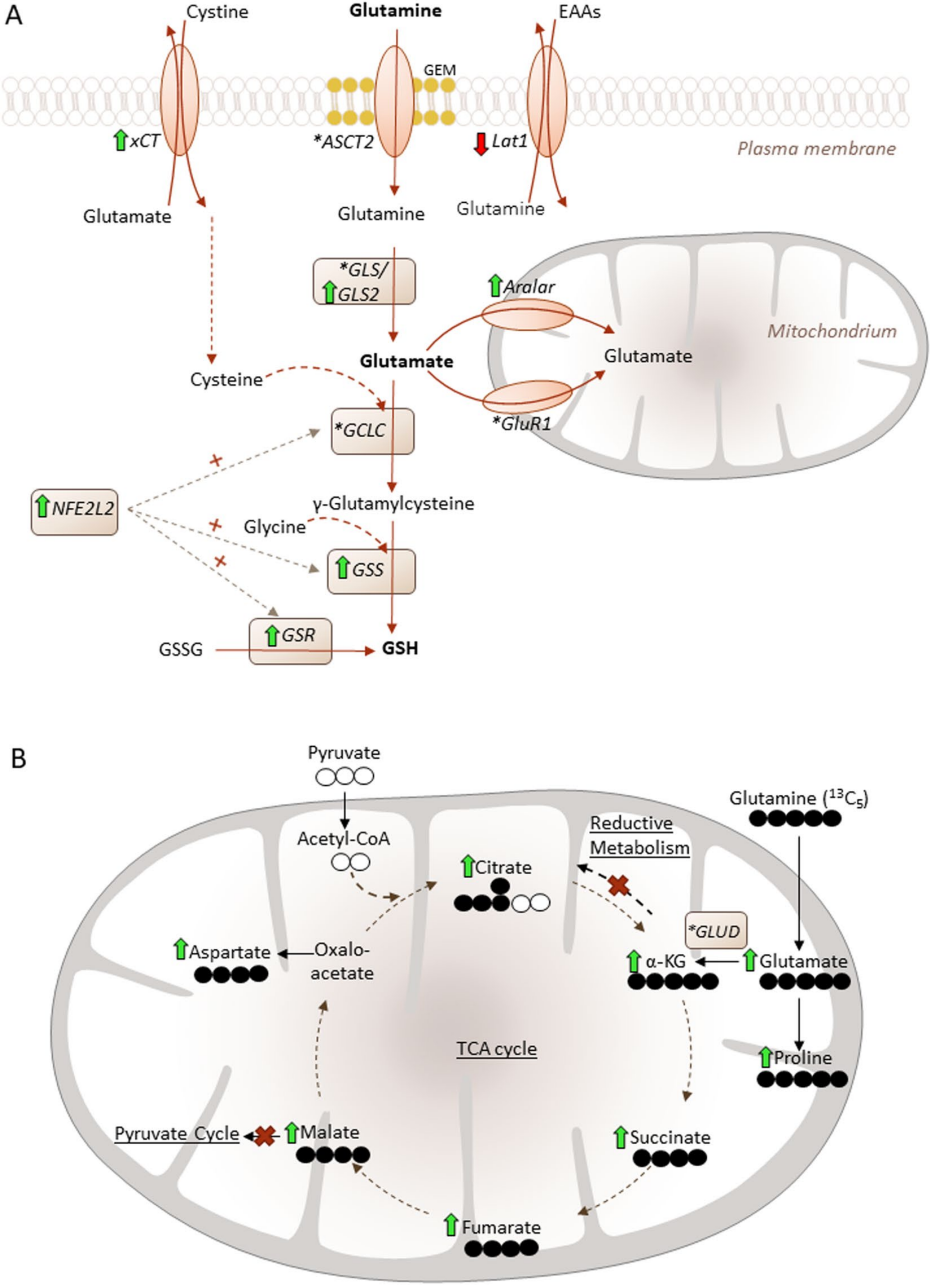
Schematic overview of the potential mechanisms of UGCG induced cellular processes. (**A**) UGCG overexpression leads to an increased intracellular glutamine concentration that fuels (**1**) glutathione synthesis via glutamate processing (green arrow = elevated mRNA level in MCF-7/UGCG OE cells as compared to MCF-7/empty cells; * = no difference; red arrow = decreased mRNA level in MCF-7/UGCG OE cells as compared to MCF-7/empty cells) (**B**) (**2**) TCA cycle turnover (green arrow = elevated metabolites analyzed by ^13^C_5_-glutamine labelling assay in MCF-7/UGCG OE cells as compared to MCF-7/empty cells) and (**3**) enhanced amino acid synthesis. EAAs = essential amino acids, GEM = glycosphingolipid-enriched microdomain, xCt = *glutamate/cysteine antiporter*, ASCT2 = *alanine-serine-cysteine transporter 2*, Lat1 = *L-type amino acid transporter 1*, GLS/GLS2 = *glutaminase*/*glutaminase 2*, GCLC = *glutamate-cysteine-ligase*, GSS = *glutathione synthase*, GSH = glutathione, GSSG = glutathione disulfide, GSR = *glutathione reductase*, NFE2L2 = *Nuclear factor (erythroid-derived 2)-like 2*, Aralar = *mitochondrial aspartate-glutamate-carrier*, GluR1 = *glutamate carrier*, *GLUD* = *glutamate dehydrogenase*.

With this study we were able to show that UGCG overexpression in breast cancer cells leads to increased mRNA expression of glutamine metabolizing enzymes resulting in an increased oxidative stress response and increased glutamine oxidation to provide sufficient energy for cell proliferation (Fig. 5).

Glucose limitation in the surrounding media has no impact on the proliferative advantage of MCF-7/UGCG OE cells, which may be ascribable to increased mRNA expression of the glucose transporter GLUT1, GLUT10 and GLUT12. Upregulation of glucose transporters allows a more efficient glucose uptake of the in the media remaining glucose into the cell. Intracellularly, the glucose seems to be used instantly for the cell metabolism, which could be the reason for a decreased glucose concentration in MCF-7/UGCG OE cells. GLUT1 upregulation is shown in several cancer types and is possibly an essential process in tumor progression^13^. GLUT1 upregulation^14^ and regulation of intracellular GLUT1 trafficking is mediated via PI3K/Akt signaling pathway^15^. Interestingly, in previous studies we could show that UGCG overexpression leads to activation of the Akt signaling pathway in breast cancer cells^4^. We could not detect any changes on GLUT1 protein level following UGCG overexpression (Supplemental Data 4), but GLUT1 activity is influenced for example by the lipid composition of the respective membrane section^16^. We also showed an altered composition of GEMs in the plasma membrane of MCF-7/UGCG OE cells, which possibly alters GLUT1 localization and activity. However, the results are not consistent with findings from Chaudhari *et al*. as they reported an increase of glucose uptake and an increase of GLUT1 protein level after inhibition of the UGCG with PPMP in the murine fibroblast cell line NIH-3T3^16^. Inhibition of UGCG expression by Miglustat treatment had no impact on GLUT1 protein level (Supplemental Data 4). GLUT10 is known to act as an intracellular transporter of ascorbic acid^17^, which is an antioxidant and could contribute to the oxidative stress response in MCF-7/UGCG OE cells (see also discussion of **1**) further down). The in MCF-7 cells important GLUT12^18^ is shown to be regulated in a p53-dependent manner^19^. Zawacka-Pankau *et al*. used RITA (2,5-bis(5-hydroxymethyl-2-thienyl)-furan) to activate p53, which then binds to GLUT12 promoter and represses promoter activity^19^. Since MCF-7 cells express wildtype p53, it would be interesting to investigate the influence of UGCG overexpression on p53 activity and a potential effect on GLUT12 expression. GLUT8 mediates apoptotic events induced by trehalose, a disaccharide derived from fungi^20^. Possibly, other stimuli induce autophagy via GLUT8 as well and therefore MCF-7/UGCG OE cells downregulate GLUT8 mRNA expression. We could not detect any difference in autophagic processes following UGCG overexpression (Supplemental Data 5).

In contrast to glucose, the glutamine concentration is essential for the proliferation advantage of MCF-7/UGCG OE cells as compared to control cells. The slightly, but not significantly increased intracellular glutamine concentration could be achieved by either increased glutamine uptake or increased *glutamate-ammonia ligase*, which uses glutamate for glutamine production. Our proliferation assay data under glutamine depletion show that the glutamine supply depends on the extracellular glutamine pool. It is shown that the stabilization of ASCT2 in the membrane is mediated by the GTPase *Rho*^21^. Accordingly, it would be interesting to check activated *Rho* in MCF-7/UGCG OE cells. Furthermore, Zama *et al*. showed that ASCT2 localizes in sphingomyelin-enriched domains together with Lat1^22^. The glutamine seems to be used in the cell for three different purposes. (**1)** glutamate production and subsequently GSH production for ROS defense, (**2)** fueling TCA cycle and (**3)** possibly proline synthesis to promote cell proliferation. (**1)** The increased proliferation and increased anabolic processes following UGCG overexpression^4^ leads to an induced oxidative stress response, which is indicated by increased mRNA expression of several glutamine metabolizing enzymes, which results in GSH production (Fig. 5A). Since total ROS levels are decreased in MCF-7/UGCG OE as compared to control cells, we conclude that UGCG overexpression leads to increased ROS defense mechanisms. This is also shown by less sensitivity of MCF-7/UGCG OE cells against TBHP, increased total antioxidant capacity and increased GSH/GSSG ratio. Chan *et al*. showed that C_6:0_-ceramide induced apoptosis is mediated via ROS in the breast cancer cell line MDA-MB 435^23^. Treatment with the UGCG inhibitor PPMP potentiated the effect^23^. Interestingly, apoptosis induction was inhibited by GSH. Our results are in line with these data, because an UGCG overexpression leads to a glutamine derived increase of GSH concentration and previously we showed increased mRNA expression of anti-apoptotic genes^4^. Glutamine-derived glutamate supports GSH biosynthesis also by increasing cystine influx by the xCT. The cysteine/glutamate exchange mediated by xCT is essential for maintaining intracellular GSH levels^24^. Furthermore, xCT, whose mRNA expression is increased in MCF-7/UGCG OE cells, contributes to breast cancer progression^25^. UGCG activity leads to the globo-series *globotriaosylceramide* (Gb3)-mediated activation of c-Src/β-catenin signaling and results in maintenance of breast cancer stem cell properties^26^. Furthermore, Kato *et al*. showed that CD44v stabilizes xCT leading to reduced oxidative stress (reviewed in^27^), whereas xCT inhibition leads to increased cellular ROS levels and suppresses tumor growth^25^. Since Lat1 transports branched side-chain amino acids such as L-leucine into cells in exchange for the efflux of intracellular amino acids such as glutamine (reviewed in^28^), it is consequential that MCF-7/UGCG OE cells exhibit a decreased mRNA expression of Lat1 (Fig. 5A). However, the Lat1 downregulation in MCF-7/UGCG OE cells is contradictory to studies from Nicklin *et al*.^29^, who showed that increased intracellular glutamine levels lead to induced Lat1 for leucine uptake. Subsequently, Lat1 activates mTORC1^29^. Possibly, decreased Lat1 mRNA expression is a counter-regulation in MCF-7/UGCG OE cells for maintaining an increased intracellular glutamine concentration. However, Lat1 is regulated by *aryl hydrocarbon receptor* (AHR) in MCF-7 cells and MCF-7 cell proliferation depends on Lat1 and AHR^30^. MCF-7/UGCG OE cells exhibit increased GLS2 mRNA expression, which is essential for glutamate production. Blocking glutaminase activity by CB-839 prevented pro-tumor activity in triple-negative breast cancer^31^. CB-839 is orally bioavailable and currently subject of clinical trials (discussed in^32^). We confirmed our *in vitro* results in 3D spheroids derived from MCF-7/empty and MCF-7/UGCG OE cells. 3D spheroids are comparable to *in vivo* conditions (Supplemental Data 6). Slight differences between monolayer culture and 3D spheroids could be due to the fact that the GSH content peaks in 3D spheroids at a different time point as compared to monolayer culture^33^. (**2)** The ^13^C_5_-glutamine tracer assay results indicate that glutamine contributes to TCA cycle turnover by being extensively oxidized following UGCG overexpression. This is shown by increased levels of glutamate (m + 5), α-KG (m + 5), succinate (m + 4) (p = 0.05), fumarate (m + 4), malate (m + 4) and citrate (m + 4) in MCF-7/UGCG OE cells. Notably, mitochondrial abnormalities influence the effectiveness of energy production through oxidative phosphorylation (reviewed in^34^). When oxidative phosphorylation is impaired, the mitochondrial F0-F1 ATP synthase maintains a moderate membrane potential by operating in reverse at the expense of ATP hydrolysis. In addition, expression of *pyruvate kinase M2* (PKM2) results in reduced ATP production from glycolysis (reviewed in^34^). To provide ATP despite impaired oxidative phosphorylation and glycolysis it is postulated that succinate-CoA ligase reaction in the TCA cycle may contribute to ATP level through mitochondrial substrate level phosphorylation (succinyl-CoA − > succinate + ATP) (reviewed in^34^). However, PKM mRNA expression is not altered following UGCG overexpression (Supplemental Data **7**). In addition, our ^14^C-glutamine tracer assay results indicate that following UGCG overexpression glutamine oxidation is increased as compared to control cells which is shown by increased ^14^CO_2_ levels (Supplemental Data 8). Accordingly, oxidative phosphorylation is not impaired, but even increased in MCF-7/UGCG OE cells. Despite the increase in glutamine-derived glutamate, GLUD mRNA expression is unchanged in MCF-7/UGCG OE cells (Fig. 5B). Since NADPH levels and glutamine-derived α-KG levels are increased following UGCG overexpression, we assume that the GLUD is more active in MCF-7/UGCG OE cells^35^. It is possible that NADPH is used for cellular stress response as well, but the NADPH/NADP^+^ ratio is unaltered. The ^13^C_5_-glutamine tracer data indicate that citrate derived from glutamine oxidation is not used for fatty acid synthesis following UGCG overexpression, which is consistent with increased ACC phosphorylation (ACC inactivated) and with reports of low amounts of stabilized *hypoxia-inducible factor 1α* (HIF1α) in MCF-7 cells grown under normoxic conditions^36^. Furthermore, our data indicate that UGCG overexpression in MCF-7 cells does not induce reductive carboxylation through a hypoxia-independent pathway. In addition, malate is not shuttled into the pyruvate cycle for lactate production, which is also executed only under hypoxic conditions. Glutamine fueled TCA cycle turnover leads to increased NADH levels following UGCG overexpression. NADH is presumably fed into the oxidative phosphorylation leading to increased ROS levels. The redox-sensitive E3 ubiquitin ligase substrate adaptor *Kelch-like ECH-associated protein 1* (Keap1) is oxidized and thereby inactivated following oxidative stress induction (reviewed in^37^). This leads to NFE2L2 stabilization and translocation into the nucleus. NFE2L2 regulates gene transcription of the GCLC, GSS and GSR via the PI3K/Akt signaling pathway^37^ and also other antioxidant proteins (reviewed in^38^). (**3)** The increased uptake of glutamine in MCF-7/UGCG OE cells is used for glutamate production, which is further metabolized to aspartate (Fig. 5B). It is shown that aspartate is essential for cancer cell proliferation^39^. Glutamine derived glutamate is used for proline synthesis in MCF-7/UGCG OE cells (Fig. 5B). The increased proline biosynthesis possibly contributes to the growth of MCF-7/UGCG OE cells^40^. Furthermore, it is shown that the MYC-dependent proline synthesis derived from glutamine influences glycolysis, which is shown by *extracellular acidification rate* (ECAR) data of P493 cells^40^.

Our data indicate that the UGCG has severe impacts on breast cancer energy metabolism. Augmented glutamine uptake is used for reinforced oxidative stress response, energy production and anti-apoptotic events shown by increased proline levels. The in our study revealed mechanisms give new insights into the important role of UGCG in cell energy metabolism and may contribute to better understanding of cancer cell adaption to poor nutritional supply. In addition, targeting the TCA cycle in the context of an UGCG overexpression could be a novel therapeutic approach.

## Supplementary information

Supplementary Dataset 1
