# Supplementary material for: UGCG influences glutamine metabolism of breast cancer cells
Source: Sci Rep. 2019 Oct 30;9:15665. doi: 10.1038/s41598-019-52169-7 (PMC6821892; doi:10.1038/s41598-019-52169-7)
Supplement: Supplementary file 1 — Supplementary Dataset 1 [file 41598_2019_52169_MOESM1_ESM.pdf]

UGCG influences glutamine metabolism of breast cancer cells

Nina Schömel[1], Sarah E. Hancock[2], Lisa Gruber[1], Ellen M. Olzomer[3], Frances L. Byrne[3], Divya Shah[3], Kyle L. Hoehn[3], Nigel Turner[2], Sabine Grösch[1], Gerd Geisslinger[1,4], Marthe-Susanna Wegner[1]

[1]*pharmazentrum frankfurt*/ZAFES, Institute of Clinical Pharmacology, Johann Wolfgang Goethe University, Theodor Stern-Kai 7, 60590 Frankfurt am Main, Germany.

[2]School of Medical Sciences, University of New South Wales, Sydney, New South Wales 2052, Australia.

[3]School of Biotechnology and Biomolecular Sciences, University of New South Wales, Sydney, New South Wales 2052, Australia.

[4]Fraunhofer Institute for Molecular Biology and Applied Ecology IME, Project Group Translational Medicine and Pharmacology (TMP), Theodor Stern-Kai 7, 60590 Frankfurt am Main, Germany.

Supplemental data 1

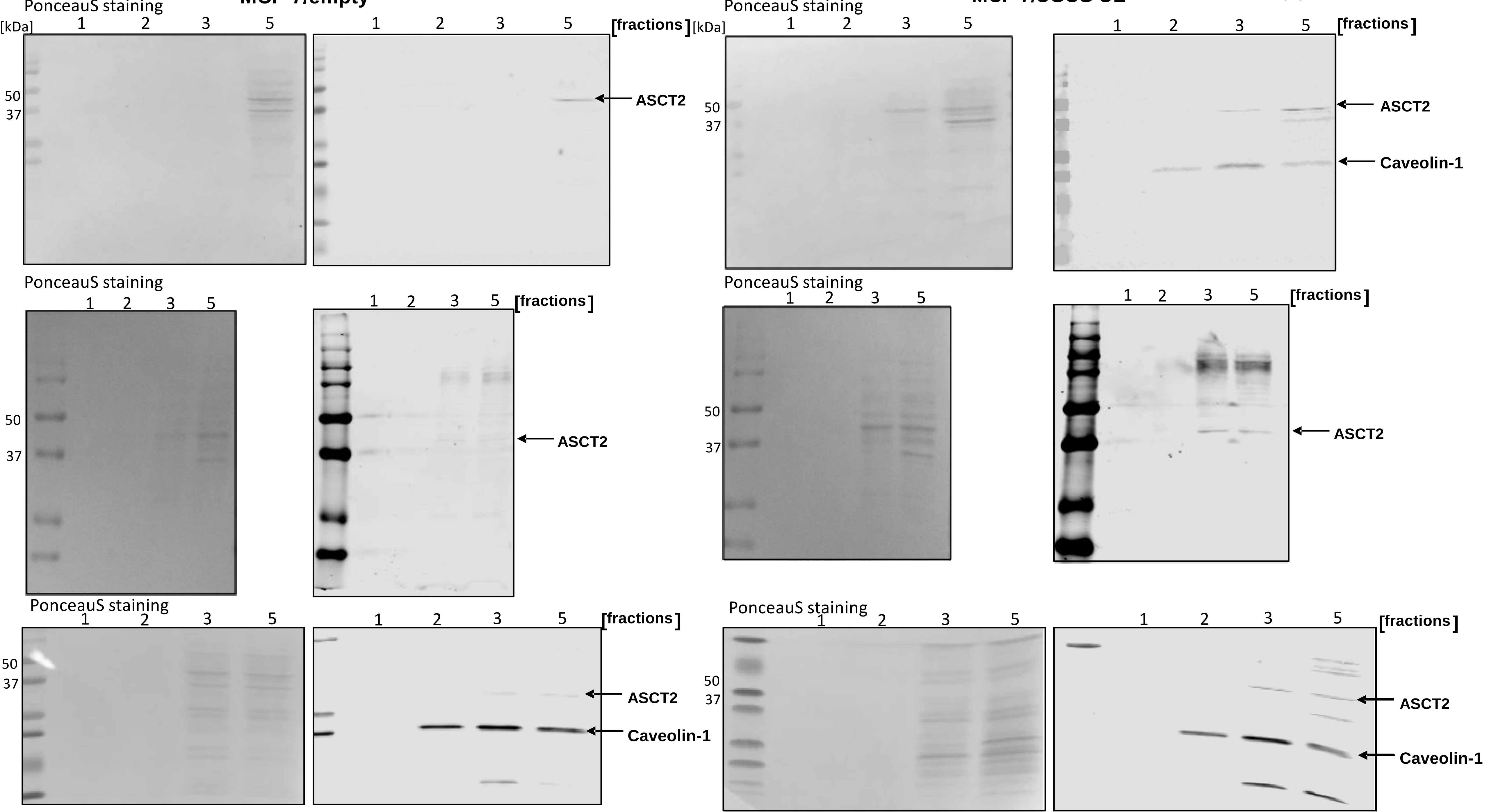

Supplemental data 2

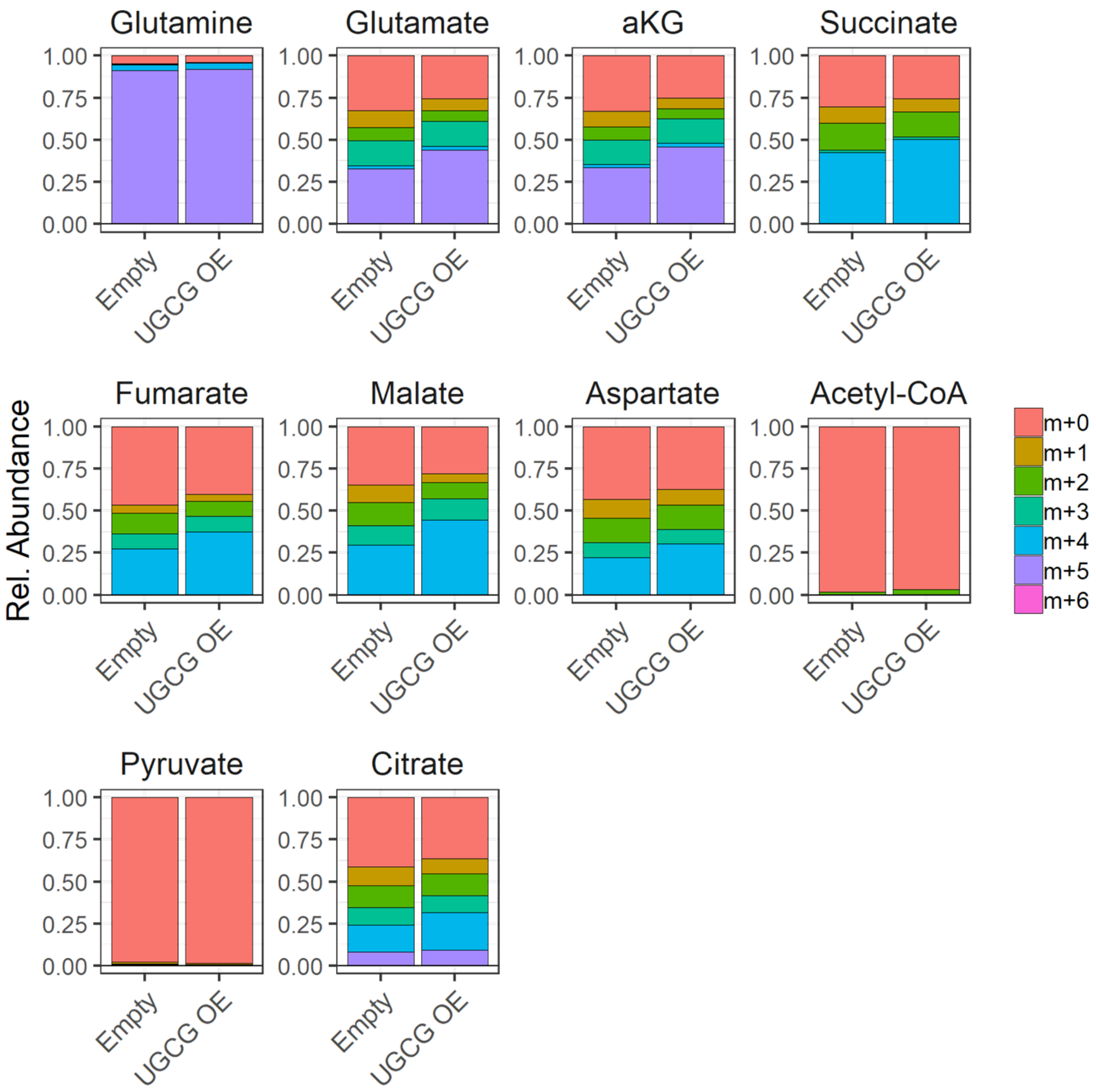

UGCG overexpression impact on glutamine oxidation. Ions distribution following $^{13}C_5$-glutamine supplementation for 16 hours in MCF-7/empty and MCF-7/UGCG OE cells.

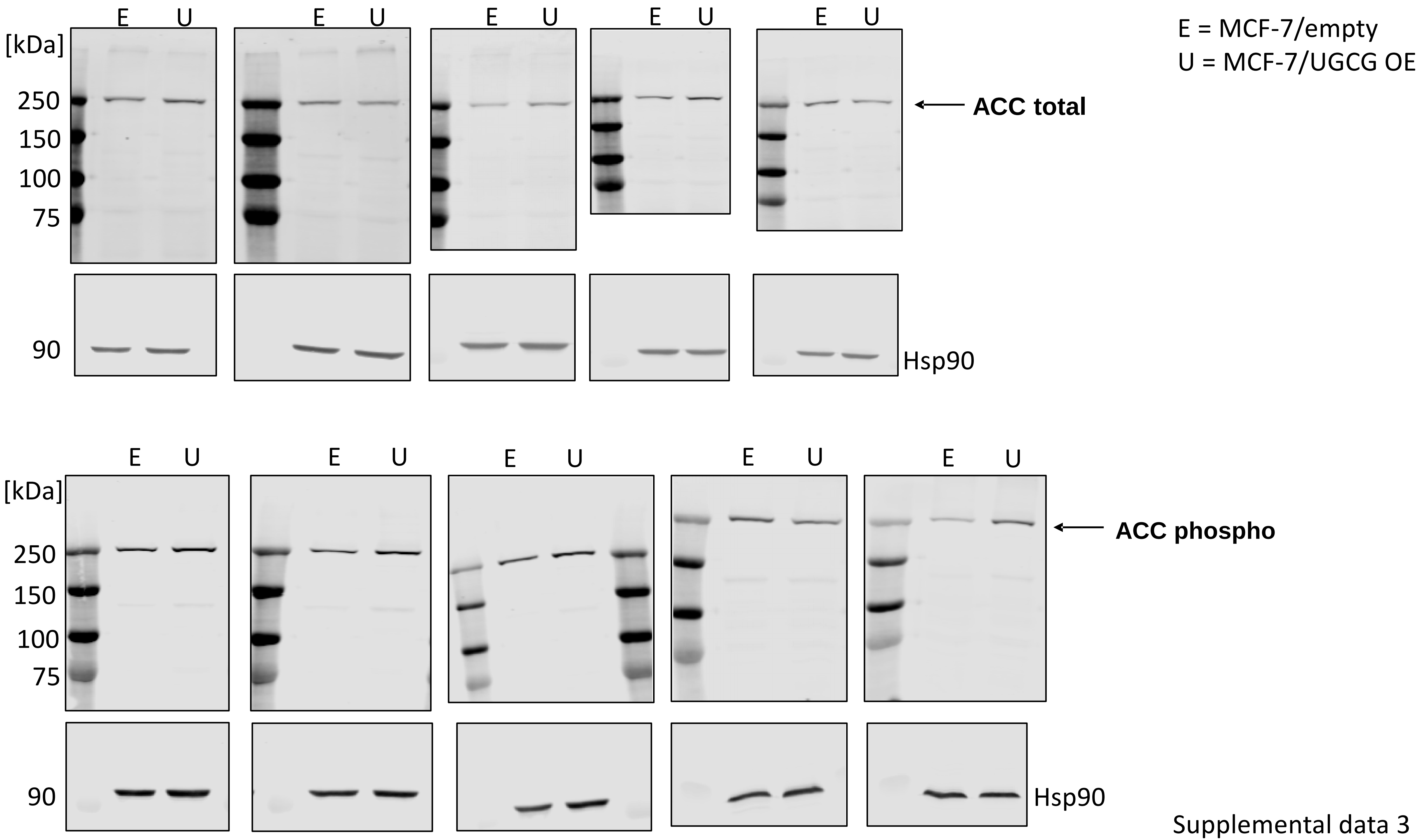

Supplemental data 3

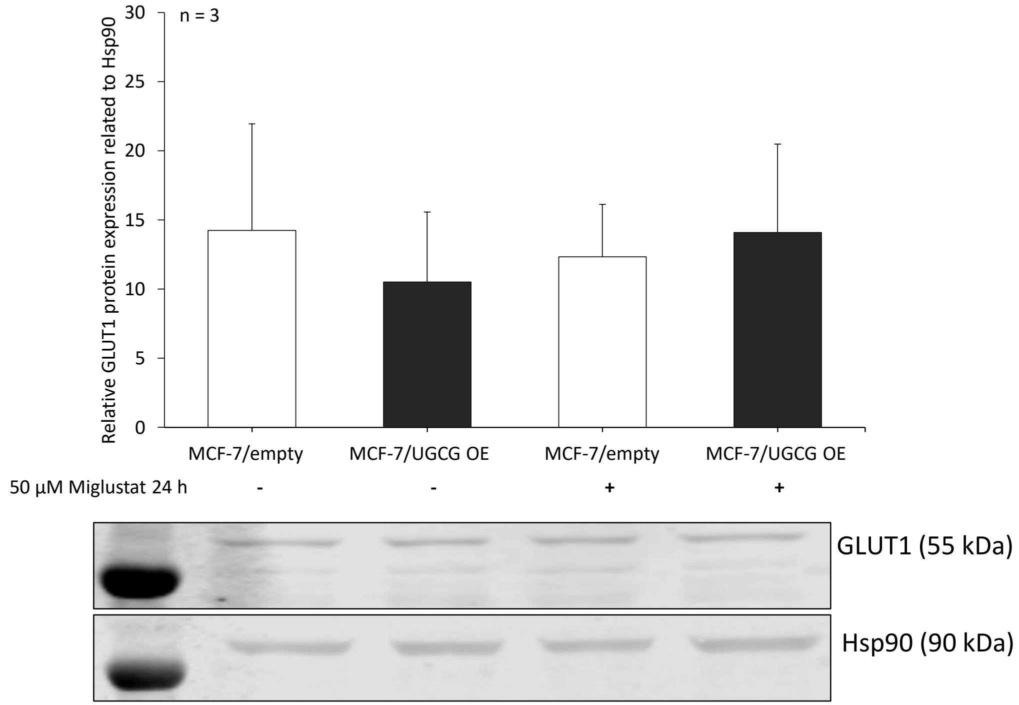

GLUT1 protein levels are unchanged in UGCG overexpressing MCF-7 whole cell lysate. A Investigation of GLUT1 protein levels by Western blot analysis. (Control and treatment with 50 µM Miglustat for 24 h to inhibit UGCG activity). The protein expression is related to the housekeeping protein Hsp90. Data are presented as a mean of n=3 ± SEM. One respective blot is displayed.

Material and Methods: Analysis of GLUT1 protein expression by Western blot. For the analysis of GLUT1 levels, cells were treated with the UGCG inhibitor Miglustat (50 µM, 24 h). Untreated and Miglustat treated cells were harvested and resuspended in PhosphoSafe^{TM} buffer (EMD Chemicals, Inc. La Jolla, USA) supplemented with 2 mM DTT (AppliChem GmbH, Darmstadt, Germany) and 1x Roche Complete (Roche, Mannheim, Germany), pH 7.4. After sonication and centrifugation, (14,000 x g, 10 min, 4 °C), the total protein concentration was determined by Bradford method. Subsequent steps were performed as described in chapter 8. GLUT1 antibody was purchased from Santa Cruz (GLUT1(A-4): sc-377228, Dallas, USA).

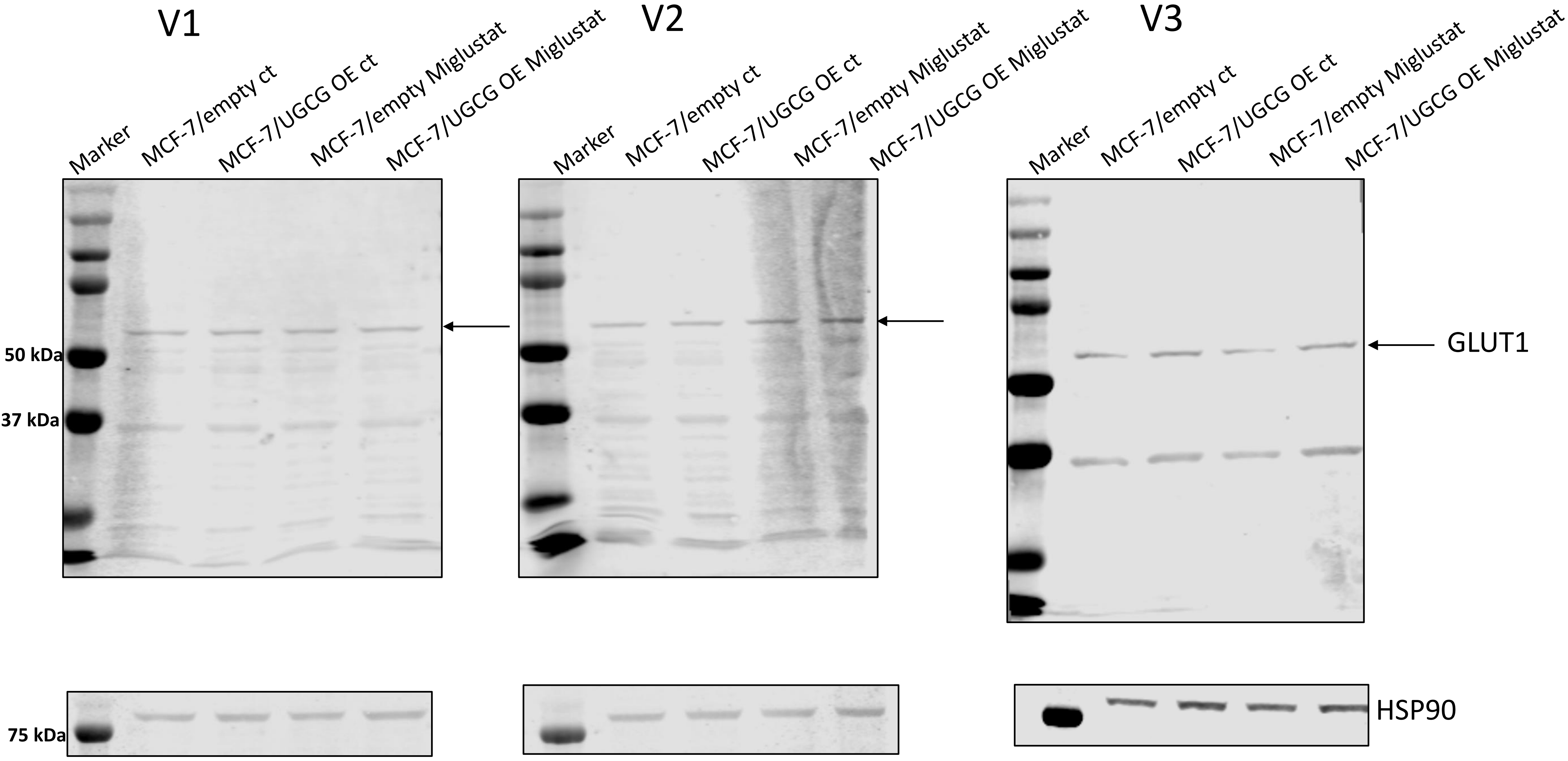

Supplemental data 4

Supplemental data 5

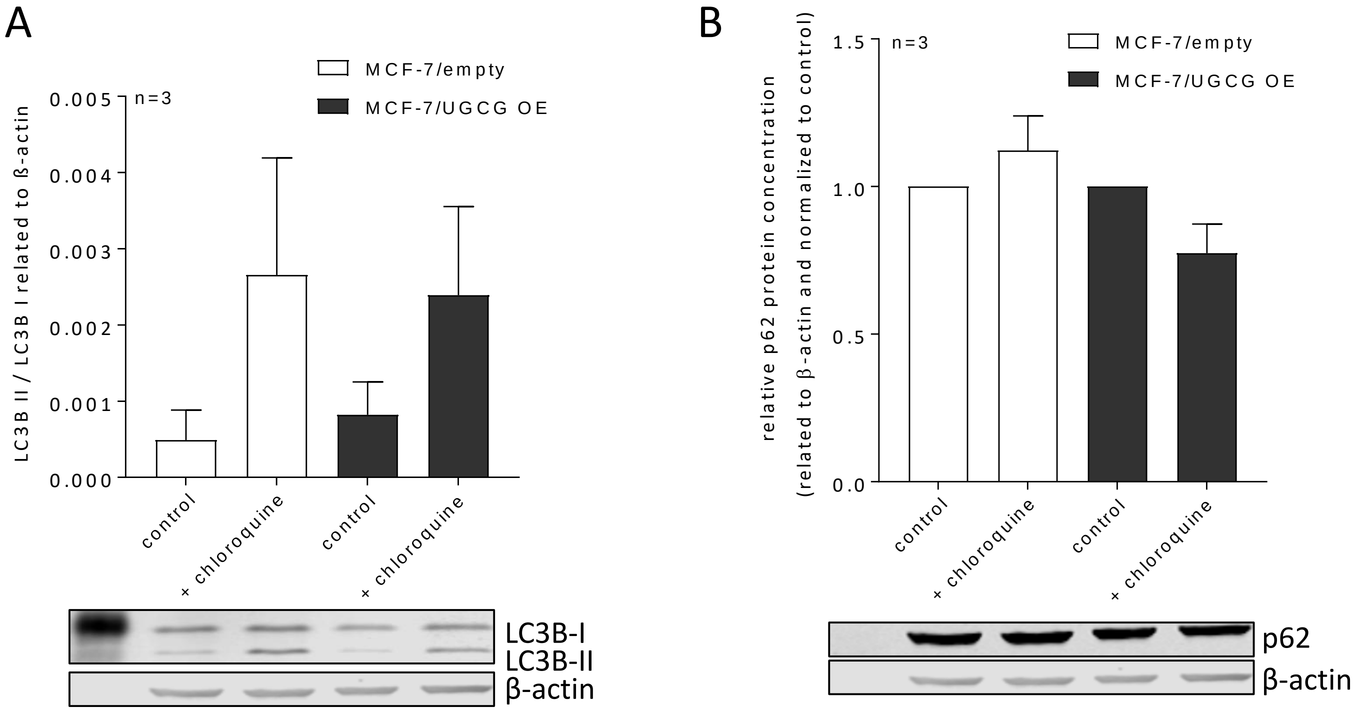

UGCG overexpression impact on autophagy induction. A Investigation of autophagic processes by determination of LC3B II/LC3B I ratio by Western blot analysis (control and 20 µM chloroquine). The protein expression is related to the housekeeping protein β-actin. Data are presented as a mean of n=3 ± SEM. One respective blot is displayed. **B** Analysis of p62 protein levels by Western blot analysis (control and 20 µM chloroquine). The protein expression is related to the housekeeping protein β-actin. Data are represented as a mean of n=3 ± SEM.

Material and Methods: Analysis of autophagy induction by Western blot analysis. For the analysis of autophagy, cells were treated with 20 µM chloroquine for four hours to block autophagic flux. Untreated and chloroquine treated cells were harvested and resuspended in PhosphoSafe™ buffer (EMD Chemicals, Inc. La Jolla, USA) supplemented with 2 mM DTT (AppliChem GmbH, Darmstadt, Germany) and 1x Roche Complete (Roche, Mannheim, Germany), pH 7.4. After sonication and centrifugation, (14,000 x g, 10 min, 4 °C), the total protein concentration was determined by Bradford method. 40 µg protein were separated electrophoretically by 12 % SDS-PAGE and then transferred onto nitrocellulose membranes. Blocking of the membrane was performed with Odyssey blocking reagent (LI-COR Biosciences, Bad Homburg, Germany) diluted 1:1 in PBST (90 minutes, room temperature). The primary antibody anti-LC3B (1:1000; rabbit polyclonal, #2775, Cell Signaling Technology, Danvers, USA) was incubated overnight at 4 °C, the anti-rabbit IRDye®680-conjugated secondary antibody (LI-COR Biosciences, Bad Homburg, Germany) was diluted 1:10,000 in blocking solution and incubated for one hour at room temperature. β-actin served as loading control (anti-β-actin (mouse monoclonal, AC-15, #A5441, Sigma-Aldrich, Deisdenhofen, Germany), 1:1000; 30 min at room temperature. Secondary antibody: anti-mouse IRDye®800-conjugated (LI-COR Biosciences, Bad Homburg, Germany), diluted 1:10,000 in blocking solution, 1 h at room temperature). Anti-p62 (mouse monoclonal, ab56416 Abcam, Cambridge, UK) was diluted 1:500 and incubated at 4 °C over night. Anti-mouse IRDye®680 (LI-COR Biosciences, Bad Homburg, Germany; 1:10,000 in blocking solution, 90 min, room temperature) served as secondary antibody. Densitometric analysis of the blots was performed with Image Studio Lite Software (LI-COR, Biosciences, Bad Homburg, Germany).

Supplemental data 6

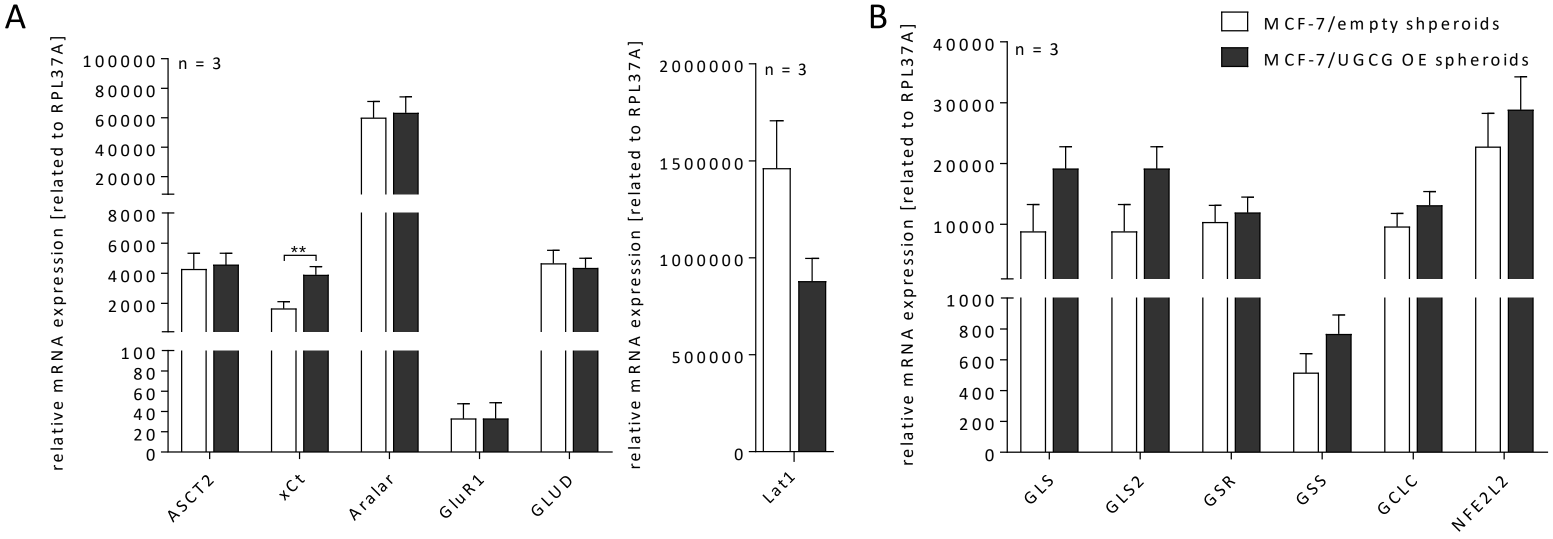

Analysis of mRNA expression of glutamine metabolizing enzymes and transporters by qRT-PCR in 3D-spheroids of MCF-7 cells. A mRNA expression analysis of the transporters alanine-serine-cysteine transporter 2 (ASCT2), glutamate/cystine antiporter (xCT), mitochondrial aspartate-glutamate-carrier (Aralar), glutamate carrier (GluR1), glutamate dehydrogenase (GLUD) and B L-type amino acid transporter 1 (Lat1) related to the housekeeping gene RPL37A. Data are presented as a mean of n=3 ± SEM. Unpaired t test with Welch's correction. **B** mRNA expression analysis of the GSH synthesis key enzymes glutaminase (GLS) and GLS2, glutathione reductase (GSR), glutathione synthase (GSS), glutamate-cysteine-ligase (GCLC) and the transcription factor Nuclear factor (erythroid-derived 2)-like 2 (NFE2L2) related to the housekeeping gene RPL37A. Data are presented as a mean of n=3 ± SEM. Unpaired t test with Welch's correction. **p≤0.01.

Material and Methods: Culture and of 3D-spheroids.

For mRNA analysis, about 120 spheroids of MCF-7/empty and MCF-7/UGCG OE cells were grown. For each spheroid, 5000 cells were seeded in 150 µl media in 1.5 % agarose-precoated wells of a 96-well plate. After seeding, the 96-well plates were centrifuged (5000 rcf, 5 min, room temperature) and subsequently incubated for four days at 37 °C and 5 % CO_2. Spheroids were harvested with a low-binding pipette tip and transferred into a reaction tube on ice. After washing with 1 x DPBS, spheroids were lysed using accutase solution (Sigma-Aldrich, St. Louis, Missouri, USA) (30 min incubation at 37 °C). Centrifugation at 500 rcf for 5 minutes pellets the cells, which were proceed as described in chapter 6.

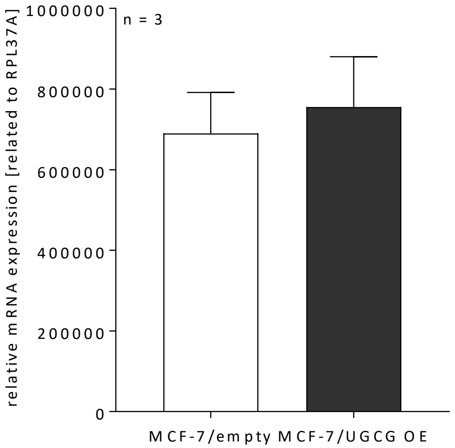

Influence of UGCG overexpression on PKM mRNA expression. The mRNA expression of the pyruvate kinase M1/2 (PKM) is related to the housekeeping gene RPL37A. Data are presented as a mean of n = 3 ± SEM.

Material and Methods: qRT-PCR analysis was conducted as described in chapter 6. Primers (Forward 5' → 3': cgcctggacattgattca , Reverse 5' → 3': gttcagacgagccacatt, 132 bp amplicon length) were purchased from Eurofins (Luxembourg, Luxembourg).

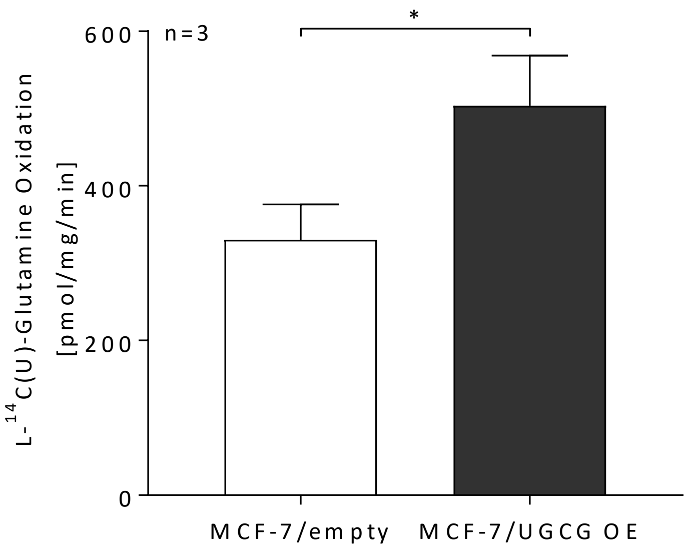

Influence of UGCG overexpression on glutamine oxidation. ^{14}C-glutamine oxidation was measured capturing $^{14}CO_2$. Data are presented as a mean of n = 3 ± SEM.

Material and Methods: Substrate Competition Assay.
Tracer measurements were performed as described in *Metabolic Vulnerabilities in Endometrial Cancer Frances L. Byrne1,2, Ivan K.H. Poon3,4, Susan C. Modesitt5, Jose L. Tomsig1, Jenny D.Y. Chow1, Marin E. Healy1, William D. Baker5, Kristen A. Atkins6, Johnathan M. Lancaster7, Douglas C. Marchion7, Kelle H. Moley8, Kodi S. Ravichandran3,9, Jill K. Slack-Davis3,10, and Kyle L. Hoehn1,2,10,11*. Briefly, 1.5 x 10^4 cells/24 well were seeded and incubated in Krebs Ringer Phosphate (KRP) nutrient buffer containing L[14C(U)]-glutamic acid. To quantify glutamine oxidation, media was acidified and evolved $^{14}CO_2$ trapped via reaction with 0.1 ml KOH before liquid scintillation spectrometry. To quantify tracer incorporation into cellular lipids, a chloroform–methanol (2:1 vol./vol.) extraction was performed and fractions assayed by scintillation spectrometry.
